# The Contingency of Frailty Level with the Perception of Risks of Falls in Various Living and Public Environments of Older Adults

**DOI:** 10.3390/healthcare13243234

**Published:** 2025-12-10

**Authors:** Snježana Brući, David Bogataj

**Affiliations:** 1 Department of Social Gerontology, Alma Mater Europaea University, 2000 Maribor, Slovenia; snjezana.bruci@almamater.si; 2 Department of Nursing, University of Applied Sciences Ivanić Grad, Moslavačka 13, 10310 Ivanić Grad, Croatia; 3 Institute INRISK, Kidriceva 1, 8210 Trebnje, Slovenia

**Keywords:** frailty, risk perception, older adults, residential environment, public space, Edmonton Frail Scale, mobility, fall prevention

## Abstract

**Aim**: This study investigated the association between frailty levels as determined by the Edmonton Frail Scale and the prevalence of perception of the risk of adverse falls in the domestic and outdoor environment among those older adults who fell so hard that they required hospital treatment (H.) and those older adults who had never encountered such a problem related to falls (C.). Predisposing and triggering factors for falls can be controlled, which is of interest from a public health perspective and, therefore, studied here. **Methods**: A quantitative cross-sectional study was conducted on a sample of 400 pensioners over 65 years of age from Zagreb region (1.2 million inhabitants, and more than 20% are aged 65+), which involved dividing the population into two subgroups: those who had fallen so hard that they had required hospital treatment (here marked “from Hospital”, or H.) and those who had never encountered such a problem related to falls and are registered as a pensioners in the region (here marked “from Community” or C.). The Edmonton Frail Scale and an additional questionnaire were used to assess the impact of frailty on perceptions of the risk of adverse falls in specific areas of their living environment. Data were collected by nurses using a structured questionnaire face-to-face, and a Chi-square test was used to analyze the dependency, while the z + 4 test evaluated the differences in percentage of those who are aware of a fall risk in case of moderate frailty or severe frailty among these two subgroups. **Results**: A statistically significant dependency was found between the degree of frailty and the prevalence of the perception of fall risk at least in one of the subgroups H. or C., especially in their homes, moving around their home, climbing up and down stairs, and in the kitchen. Comparing the prevalence of perception of the risk between C. and H., the difference in recognition of risk hazard has been significant for climbing up and down stairs. Among the major problems of non-adapted buildings are slippery floors (for C.), poorly installed or no fence at all (for H.), and inadequate or poor lighting (for H.). The perception of external obstacles (sidewalks, distance from institutions, public lighting) also increases with the deterioration of the functional state. **Conclusions**: The results confirm the importance of individualized adaptation of residential and public spaces in accordance with the degree of frailty. It is necessary to warn older adults about the risks of falling, both those who have not yet had this experience, as well as those who have suffered the consequences that have led to hospitalization, to reduce the frequency and consequences of falls.

## 1. Introduction

Frailty, defined as a syndrome of gradual decline in physiological reserves and functionality of several organ systems, increases the vulnerability of older adults to adverse health outcomes, particularly adverse falls [1]. The prevalence of this syndrome among the older adults in the community reaches up to 70%, indicating its prevalence and importance as a public health priority [2]. Falls are a common consequence of frailty and one of the main reasons for hospitalization of older adults. The current health system response is still largely based on reactive measures rather than proactive prevention [3]. The risk of falls is further increased in the presence of sarcopenia, malnutrition, and several chronic diseases that often accompany frailty [4]. After a fall, as many as 88.5% of older people develop a fear of falling again, which can lead to reduced mobility, depression, and further deterioration of functional ability, creating a vicious cycle of frailty deterioration [5]. People with frailty are significantly more likely to develop fractures, especially hip fractures, which almost always require hospitalization and pose a long-term threat to independence and quality of life [6,7]. In such cases, the functional reserve is further reduced, and the progression of frailty accelerates, especially if the person is facing a loss of autonomy or institutionalization [8]. Ageing is a natural process that is accompanied by systemic changes, including loss of muscle mass, flexibility, and functionality of the nervous system, which, together with the presence of frailty, increases the risk of falls [9]. Such events often result in serious consequences, including irreversible decline in functional abilities and increased mortality [10]. Given the multifactorial, dynamic nature of frailty development, which involves the interaction of physiological, psychological, and environmental factors, it is imperative to develop integrated and targeted interventions to prevent falls and maintain the functionality of older adults [11,12]. Falls often represent the first step towards a loss of independence. The link between frailty and the risk of falling further complicates the day-to-day functioning in the home environment and in the community. Identifying and understanding the exposure to risks and safety factors that affect falls in the context of different levels of frailty is critical to developing effective prevention strategies. Given the growing demographic representation of the older population, especially in European countries with a growing proportion of people aged 65 and over, the need to understand the interconnection between physical fitness and the living environment is becoming a priority. Falls affect not only the health system with increased treatment costs, but also the psychosocial state of older adults, who reduce the level of activity and social inclusion due to the fear of falling again.

### 1.1. Frailty and Undesirable Decline

Therefore, frailty-related falls pose significant challenges to modern health systems [13,14,15]. Decreased muscle strength and body mass, high levels of fear of falling, and consequent slowing of walking speed contribute significantly to the development of frailty and, thus, to the more frequent occurrence of falls [16,17]. Fear of falling is associated with reduced performance in multitasking activities and a reduced ability to perform daily tasks. Although directly related to a person’s current condition, some studies show that the prevalence of falls increases with age. People with frailty are attributed a 59% higher incidence of falls compared to their non-frail peers [18]. It is recognized that falls are causally related to natural changes occurring in the body that are characteristic of the ageing process, e.g., impaired balance, decreased visual acuity, mobility, and functional ability, which are additionally associated with frailty [19,20]. Frailty also increases an increased risk of a decline in metabolic diseases, e.g., diabetes [21] and hypertension [22], which implies the need to change medication treatment, including the number and type of medications that older people with frailty take regularly. Early diagnosis at an early stage of frailty is not an expensive procedure and should, therefore, be part of standard practice to allow for the timely selection of an appropriate therapeutic regimen and reduce the risk of falls and hospitalization [23]. Falls are the second most common cause of death due to accidental injuries, with an estimated 37.3 million people who fall needing medical attention each year, generating significant costs [24]. Older people, especially women, represent a group at high risk of falling, and falls can lead to permanent disability and the need for institutional care. The ageing process leads to changes such as joint deformities, decreased reflexes, or the inability to maintain balance [25,26]. Approximately 30% of people over 65 years of age have difficulty walking, and 20% use assistive devices [27]. Additional risks include visual impairment, orthostatic hypotension, heart and lung disease, and polypharmacy [25]. Physical environmental factors (unevenness, poor lighting, obstacles in the house) are present in 30–50% of fall cases and are often associated with poverty, which prevents better adaptation to the environment or access to assistance [28,29]. Despite numerous studies, predicting an individual’s risk of falling remains a complex challenge [30]. Declines are not due to a single factor, but to a complex interaction between many risk factors, and the risk increases linearly with their number [31,32,33]. The question arises as to how older people perceive their exposure to risk and whether this is related to their functional capabilities and previous experiences of falls. Therefore, the question also arises as to whether older adults who have already been hospitalized due to a fall are more often aware of their exposure to fall risks.

### 1.2. The Role of the Built Environment and Smart Solutions in Preventing Falls: Linking Technology and Infrastructure

The built environment plays a key role in reducing the risk of falls, especially in rural areas facing an accelerating ageing population [34,35]. Older adults often suffer from physical weakness and cognitive decline, resulting in reduced mobility and increasing susceptibility to falls and other accidents [34]. Unsuitable residential and public spaces, such as those without ramps, with poor lighting, or slippery floors, further increase the risk. Smart villages and retirement communities [35] with integrated cyber-physical systems (CPS) offer innovative solutions based on the Internet of Things (IoT), ambient intelligence, sensors, and cloud computing [35]. Such systems allow for continuous monitoring, identification of behavioural patterns, and timely intervention in case of risk [35,36]. Ambient technologies embedded in smart buildings support the autonomy of the older adults, reducing the need for institutionalization and the risk of falling [37,38]. In addition to technology, spatial planning must be based on the concept of an “age-friendly environment”, by removing architectural barriers and creating safe, accessible, and easily navigable spaces [39]. The collaboration of experts from various fields, including architecture, healthcare, social care, logistics, ICT, and construction, is crucial for the holistic development of these environments [40]. Managing the built environment through facility management (FM) further strengthens the social aspect of planning [35]. FM enables interactive communication between users and systems and the co-creation of solutions that meet the real needs of older residents [41]. Such an approach not only creates a safer physical environment but also strengthens social cohesion, reduces isolation, and improves quality of life [42].

### 1.3. Research Questions and Hypotheses

Based on the literature cited above and the experiences when we were working with the population 65+ in the hospital and outside the hospital, the following research questions have been formulated:

RQ1: Is there any association between frailty levels as determined by the Edmonton Frail Scale and the perception of the risk of adverse falls in the domestic and outdoor environment for the population of those who experienced falls so hard that they required hospital treatment, and those older adults who had never encountered such a problem related to falls?

RQ2: Are there any differences in perception of the risk of adverse falls in the domestic and outdoor environment between those who fell so hard that they required hospital treatment and those older adults who had never encountered such a problem related to falls?

We provide the following hypotheses:

**H1.**  *The prevalence of perception of the risk of adverse falls in some situations of the movement of older adults in the domestic environment and frailty levels, as determined by the Edmonton Frail Scale, is not independent.*

**H2.**  *The prevalence of perception of the risk of adverse falls in some constructions in older adults’ homes and frailty levels, as determined by the Edmonton Frail Scale, is not independent.*

**H3.**  *The prevalence of perception of the risk of adverse falls in some situations of the outdoor environment and frailty levels, as determined by the Edmonton Frail Scale, is not independent.*

These topics have never been conducted in the country where the sample was recruited.

## 2. Materials and Methods

The study was conducted in 2024 and 2025 on a randomly chosen sample of 400 respondents over the age of 65 in the Zagreb region. Structured questionnaires, frailty assessment using the Edmonton Frail Scale, and analysis of home and community conditions were used. The research combines quantitative and qualitative elements, including descriptive analyses and inferential statistics (Chi-square test, and due to the potential weakness of the Chi-square tests, we also added the z + 4 test). Two instruments were used for the study: one instrument is a standardized questionnaire to assess frailty (Edmonton Frail Scale), and the other instrument is an additional questionnaire to assess the impact of frailty and the living environment on the perception of risk of falls. The Edmonton Frail Scale is a questionnaire that samples 10 domains; the highest score is 17 and represents the highest level of frailty. Two domains are tested using performance-based elements: the clock test [43] for cognitive impairment and the “Timed Get Up and Go” [44] for balance and mobility. Other areas include mood, functional independence, medication use, social support, nutrition, health relationships, incontinence, burden of disease, and quality of life, all standard historical items in geriatric assessment [45]. Edmonton’s fragile roster also has good structural validity, good reliability, and acceptable internal consistency [46]. A unique feature of an instrument of clinical frailty is its inclusion in the field of social support, indicating the adoption of a dynamic frailty model [47].

The additional questionnaire included the following main questions:

I. Circle “yes” or “no” in front of the following statements about situations that pose a risk of falling for you and are present in your home!
  - It is risky for me to enter or leave the home.
  - It is risky for me to move around my home.
  - It is risky for me to climb up and down stairs.
  - It is risky for me to use the kitchen.
II. Circle “yes” or “no” in front of the following statements about the conditions in your home that pose a risk of falling and are present in your home movement
  - Uneven, excessively low, or high stairs pose a risk to me.
  - Slippery floors pose a risk to me.
  - Unmarked edges (especially a raised threshold from the door) pose a risk to me.
  - A poorly installed or no fence at all poses a risk to me.
  - Inadequate or poor lighting.
III. Circle “yes” or “no” in front of the following statements about the area of your movement outside the home that might pose a risk of falling and are present in your movement. Circle “yes” if you agree with the following
  - The transport infrastructure in your area is adapted to the older adults and the disabled and does not pose a risk of falling?
  - The streets and parks you walk through in your place are sufficiently lit.
  - The sidewalk you move on is regularly maintained and not slippery or uneven.

The study included people aged 65 years and older of both sexes who live in a family or alone, who can communicate and move independently, and who have not proven a certain level of frailty or not. The exclusion criteria in the study were people under 65 years of age or with dementia or cognitive impairment, as reported by family members and recognized by family doctors. Therefore, the results, which we generalize to the entire population of the Zagreb region, do not include severely demented residents. Prior to enrolling in the study, each respondent received an information form and signed a consent form to participate. The study included 400 participants who were divided into two groups. The first 200 respondents consisted of 200 randomly chosen already hospitalized pensioners due to falling in old age (100 women and 100 men among all being already hospitalized), while the second group of 200 respondents were older pensioners with no experience of falling and having serious consequences (no hospitalization has been needed). The survey was conducted by nurses from the Zagreb region, who deal with these kinds of problems daily, face-to-face.

## 3. Results

### 3.1. Samples of Tested Respondents from the Zagreb Region

For studying differences in distributions of frailty among those who recognize a fall hazard and those who do not recognize it, in the subgroup of those who had been hospitalized (H.) and those who had not been (C.), the distribution of frailty was compared between subgroups C. and H. among those who confirmed “Yes” and “No”. We additionally studied where they are living, either in the city or in a rural area.

From Table 1 it follows that in the population of older adults aged 65 and over from the Zagreb region, we can expect that the percentage of frail individuals increases with increasing frailty category in subgroup of those who have already been hospitalized (Spearman’s correlation coefficient is 0.9), and decreases with increasing frailty category for those who have no experience with serious falls (Spearman’s correlation coefficient is 0.82).

The distribution of frailty levels among answers of those from the urban population is like the distribution of frailty among answers from the rural population. No significant differences have been detected (Table 2).

### 3.2. Distribution of Frailty Among Those Who Had Been Hospitalized and Those Who Had Not, Regarding Recognition of Fall Hazards in Their Home

The dependence between the perception of risk of falling at home and various frailty categories according to the results of the Chi-square test was studied. Therefore, the first set of questions was designed to obtain the figure of distributions regarding frailty of those who recognize a fall hazard, being present in their home (answering “yes”), and those who some situations in their home, do not recognize it as a hazard (answering “No”). They were asked to circle “Yes” or “No” when asked whether they considered themselves at risk of falling in the following situations:

- Entering or leaving the home;
- Moving around their home;
- Climbing up and down stairs;
- Using their kitchen.

Table 3 shows the distribution of frailty in dependence on their answers.

In all the older adults’ home environments listed in the first column in Table 3, except for the case of “climbing up and down stairs”, the proportion of those who do not recognize the risk in the situations is higher than the proportion of those who recognize the risk. However, when using the Chi-square test, in the following cases, we found the significance:

- “Climbing up and down stairs”, for subgroup C., significantly recognized the dependence between prevalence of risk and frailty level (*p*-value = 0.035);
- “Moving around their home”, for subgroup C. (*p*-value = 0.031) and subgroup H (*p*-value = 0.1) was significantly recognized the dependence between prevalence of risk and frailty level;
- “Using their kitchen”, for subgroup C. was significantly recognized the dependence between prevalence of risk and frailty level (*p*-value = 0.018).

Therefore, the prevalence of perception of the risk of adverse falls in these three situations of the domestic environment and frailty levels, as determined by the Edmonton Frail Scale, is not independent.

But in all cases in Table 3 and Table 4, the percentage of those being moderately frail or very frail (together) among all who consider the given environment to be risky is greater than the percentage of those who consider the same environment not risky among all who consider the given environment in the same way.

Table 4 also shows the Z + 4 tests of the differences in the percentages of perception and non-perception of risks in moderately and severely frail individuals only.

The differences in the percentages of the same answers in the subgroup of those who are moderately and severely frail are significant only

- in the case of those from the hospital, who consider moving around their home (H: *p*-value < 0.05),
- in both subgroups (C. and H.) when climbing up and down stairs (*p*-value < 0.05 for those from community-C., and *p*-value < 0.1 for those from the hospital-H.), and
- for those from the community, considering the use of their kitchen (C.: *p*-value < 0.05).

Therefore, we may conclude that in the samples, there were higher percentages of those having moderate or severe frailty who answered “Yes” and had already been hospitalized because of a fall (except the fall hazard in the kitchen). Therefore, the Agresti-Caffo differences p’(Yes)-p’(No) are positive. We may conclude that there are significant differences in cases of moving around their home for subgroup H. (*p*-value < 0.05), climbing up and down stairs for subgroup H. (*p*-value < 0.1) and C. (*p*-value < 0.05), and using their kitchen (*p*-value < 0.05). In these cases, the prevalence of perception of the risk of adverse falls in the population of moderate and severe frailty is assumed to be riskier than not. But only for entering or leaving the home, the difference in perceptions of risk between C. and H. groups was significant (*p*-value < 0.05).

To support the conclusions derived from Table 4, the dependence between the frequency of perception of risk of falling at home and various frailty categories has been studied based on the statements about the conditions of their home movement that pose a risk of falling and are present in their movements at home. Uneven, excessively low or high stairs which might pose a risk, slippery floors, unmarked edges, poorly installed or no fence at all, slippery floors, and inadequate or poor lighting, that might pose a risk to older adults were considered. The results of the survey in subgroups C. and H. are given in Table 5.

From Table 6, we can see that significant dependence between the distribution of frailty for those who recognize risk and those who do not is only in the case of slippery floor, namely for those from the community not having experience to be hospitalized (C.: *p*-value = 0.006) and poorly installed or no fence at all at those who are already hospitalized (H.: *p*-value = 0.008).

Therefore, the prevalence of perception of the risk of adverse falls and frailty levels as determined by the Edmonton Frail Scale is not independent in the cases of slippery floors (for subgroup C. only) and in the case of poorly installed or no fence at all (for subgroup C. only), significantly enough in the cases of movement in the domestic environment. But also, inadequate or poor lighting, in case of the subgroup H, those slightly higher than 0.05, seems to be significant enough (*p*-value = 0.054). Namely, in cases of inadequate, poor lighting show the contingency between perception of risk and frailty level is quite significant for those already being hospitalized.

Table 6 also presents the Z + 4 test of difference in percentage of answers: p’(Yes)-q’(No) about their movements in the environment of home regarding construction and installations discovered for the perception of moderate and severe frail older adults. The sample differences are positive everywhere except in the case of “Uneven, excessively low or high stairs” in subgroup H, which means that a larger proportion of moderate or severe frail respondents predicted a hazardous environment in the groups of people with the same answer.

From column 6 of Table 6, we may conclude the following about percentages of those who answered the same and are moderate or severe frail:

- For persons from the community–C. that there is a higher percentage of those who are aware of the risk of falls because of uneven, excessively low, or high stairs (z = 1.301; *p*-value < 0.1);
- For persons from community–C. that there is a higher percentage of those who are aware of the risk of falls because of slippery floors and stairs (z = 3.054; *p*-value < 0.002), and that for persons being hospitalized–H., there is a lower difference in percentages of those who are aware of risk and who are not, but still significant (z = 1.329; *p*-value < 0.1);
- Only for persons being hospitalized–H., unmarked edges (especially door threshold), are assessed to be significantly riskier (z = 1.545; *p*-value < 0.07). The same we can also conclude for poorly installed or no fence at all (z = 5.823; *p*-value < 0.001);
- The differences in prevalence of perception of the risk between the subgroup C. and the subgroup H. are significant in cases of the slippery floor (*p*-value < 0.03); unmarked edges (*p*-value > 0,15; poorly installed or no fence (*p*-value < 0.0001); and inadequate or poor lighting (*p*-value < 0.01).

In open-ended responses, respondents most often pointed to problems related to bathtub use (n = 5), including entering and exiting, indicating that the bathroom is a common point of risk for falling, but the numerical answers are hidden in the hazard of slippery floors and thresholds. Two of the responses were related to the uneven surfaces around the house, and one respondent expressed a sense of insecurity. One person stated that they did not see any additional obstacles. The results highlight the importance of individual assessment and adjustments, especially in bathrooms and outdoor spaces.

### 3.3. Distribution of Frailty Among Those Who Had Been Hospitalized and Those Who Had Not Been Hospitalized Regarding Recognition of Fall Hazards in the Outdoor Environment

When we studied the dependency of outdoor risk perception and the frailty level of the old inhabitants, divided into two subgroups, we obtained Table 7, Table 8, Table 9 and Table 10. The question is written in the first column of these tables. While the distribution of unawareness of the risk of fall in subgroup H., with the degree of frailty in some cases even increases, or is fairly uniform, in subgroup C., in all cases with the degree of frailty, the frequency of awareness of risk (saying “No”) even decreases.

Comparing the distribution of “Yes” responses regarding the perception of safety when going shopping or making other necessary visits to central locations, between those from the community who have not yet been hospitalized due to a fall (subgroup C.) and those who have had this unpleasant experience (subgroup H.), we can find that there is a large difference in the distributions of frailty regarding perception of safety. Those who have not been hospitalized for falls are more likely to recognize the risk of falling than those who have been hospitalized and are distributed differently in terms of frailty (*p*-value < 0.04).

There is a high significance of dependency between frailty levels as determined by the Edmonton Frail Scale and the prevalence of perception of safety in the outdoor environment for the older adults who have been hospitalized (subgroup H.) due to a dangerous fall (*p*-value < 0.000002), found when using the Chi-square test. The significance of this dependency in the subgroup C. is lower, but still *p*-value < 0.0007.

When comparing the differences in significance of dependency between frailty levels as determined by the Edmonton Frail Scale and the prevalence of perception of safety in different outdoor situations, we obtained Table 8, Table 9 and Table 10.

Comparing positive answers (Yes) between residents who were hospitalized due to a fall (H.) and those who did not have this experience (C.), we find that the Chi-square test shows a significant difference at *p*-value < 0.04.

There is also a high significance of dependency between frailty levels as determined by the Edmonton Frail Scale and the prevalence of perception of safety in the transport for the older adults who have not being hospitalized (subgroup C.) due to a prevalence of safety in transportation (*p*-value < 0.00001) determined when using the Chi-square test, but there is no significance of this dependency in the subgroup H. (*p*-value > 0.14).

When we are comparing the frequency of answers “Yes” and “No” in a group of moderate, frail, and severe frail persons, the Z + 4 test gives the following conclusions:

- Among those being moderate and severe frail who were not hospitalized, the percentage of those who answered “Yes” in the group of all the same answers is half of those who do not recognize the environment as dangerous for falls (z = 2.91, and the *p*-value for the characteristic of the differences in percentages is lower than 0.002). It means that in the total population, the significantly higher percentage of those being moderate and severe frail who are aware of the risk in transport (*p*-value < 0.002);
- But among those of moderately or severely frail who had been hospitalized, the percentage of those who answered “No” is nearly 50% higher of those who do not recognize the environment as dangerous for falls. Therefore, we may conclude for all such populations in the region that the majority of them recognize risk in transport at a significance level high enough (z = −1.71, and the *p*-value is lower than 0.05).

We have also compared opinions among those who have already been hospitalized due to falls (H.) and those who have not had this experience (C.) regarding the light on the streets and parks they move on, considering if they assume that the streets and parks are sufficiently lit. What is the perception of safety in their area? The respondents’ responses are summarized in Table 9.

In both subgroups, positive responses were more frequent; this means that, according to their opinion, more than half of the population assumes that the illumination of the streets and parks they walk through is adapted to the needs of frail people. Chi-square test of dependency shows that there is a certain dependency of prevalence of perception of hazard and frailty in the subgroup C. (*p*-value < 0.0003), and in the subgroup H. (*p*-value < 0.0065).

The distribution of frailty between C. and H. regarding perception of safety because of accurate lights in their outdoor environment is significantly different (*p*-value = 0.0002).

When we are comparing percentages of answers “Yes” and “No” of moderate frail and severe frail persons regarding the satisfactory lighting of the external environment, the Z + 4 test shows significant differences in percentages.

- Among those who were not hospitalized, the percentage of those who answered “Yes” and were moderately or severely frail is only half of those who do not recognize the environment as safe for falls (z = 2.63, and the *p*-value for the significance of the differences in percentages is lower than 0.005). It means that in the total population, the percentage of those who are aware of the risk of falling due to insufficient lighting is significantly higher.
- Also among those of moderately or severely frail who had been hospitalized, the percentage of those who answered “No” is higher than percentage of those who answered “Yes” regarding recognizing the environment as safe for falls due to lighting (z = 1.40 and the *p*-value for the significancy of the differences in percentages is 0.08). It means that in total population being hospitalized the percentage of those who are aware of the risk to fall due to insufficient lighting is significantly higher.

We have compared opinions among those who have already been hospitalized due to falls (H.) and those who have not had this experience (C.) about the sidewalk they walk on, considering if it is regularly maintained and not slippery or uneven. What is the perception of safety in their area? The respondents’ responses are summarized in Table 10.

In both subgroups, the positive responses regarding the sum of them were less frequent; this means that according to their opinion, more than half of the population assumes that the sidewalk they move on is not regularly maintained, is slippery and uneven, and, therefore, not enough adapted to the needs of frail people. Chi-square test of dependency shows that there is a certain dependency of prevalence of perception of hazard and frailty in the subgroup C. (*p*-value < 0.05), and in the subgroup H. (*p*-value < 0.00001).

The distribution of frailty between C. and H. regarding perception of safety, because the sidewalk they move on is not regularly maintained, is slippery, and uneven, is also significantly different (*p*-value = 0.0009).

When we are comparing percentages of answers “Yes” and “No” of moderate frail and severe frail persons regarding the sidewalk they move on, the Z + 4 test shows the significant differences in percentages of their perception only for those from the subgroup H.

- Among those of moderate frail and severe frail persons who were not hospitalized, the percentage of those who answered “Yes” is 27%, but “No” answered 29% of with the same answer. There are no significant differences.
- Among those of moderately or severely frail who had been hospitalized, the percentage of those who answered “No” among all of the same answer is 37%, and the percentage of those who answered “Yes” among all of the same answer regarding the sidewalk they moved on was 52%. Therefore, the difference in percentages is significant (*p*-value < 0.017).

## 4. Discussion

The results presented in Table 1 indicate the diversity of the functional status of the older adults in the Zagreb region according to the Edmonton Frail Scale. The sample is divided into subgroups of those who experienced a fall severe enough to require hospital treatment (H.) and those who did not have such experiences (C.). We can say that the proportion of frail individuals increases with increasing frailty category in the subgroup of those who have already been hospitalized and decreases with increasing frailty category for those who have no experience with serious falls. Overall, more than 43% of respondents from the subgroup C., and over 65% of those who experienced hospital treatment, H., are on the frailty spectrum: mild, moderate, and severe frail, indicating the need for targeted support and adaptation of their environment to maintain the independence and quality of life. These results confirm the importance of regular monitoring of the functional status of older people to identify individuals exposed to risk promptly and to implement measures to prevent unwanted falls. In these cases, we should also consider the development of assisted living and the use of smart technologies. The data given in this paper provide the basis for further analyses of the relationship between frailty level and the factors of the built environment that may affect the risk of falls and the need for institutionalization.

According to the results in Table 4, we may confirm the first hypothesis (H1): The prevalence of perception of the risk of adverse falls in some situations of the movement of older adults in the domestic environment and frailty levels, as determined by the Edmonton Frail Scale, are not independent.

It is especially significant for those who had not experienced a fall, or hospitalization was not needed, in case of moving inside their home (*p*-value < 0.04), but for those who had experienced a fall and hospitalization was needed, *p*-value is equal to 0.1, when confirming that prevalence of perception of the risk of adverse falls in case of moving inside their home and frailty levels as determined by the Edmonton Frail Scale are not independent. Climbing up and down stairs shows significant dependency of frailty level and prevalence of perception of hazard (*p*-value < 0.04) in the subgroup of those who had no experience of hospitalization due to a fall. Also, the prevalence of perception of the risk of adverse falls in case of being in the kitchen and frailty levels as determined by the Edmonton Frail Scale are not independent for subgroup C (*p*-value < 0.02). Therefore, the prevalence of perception of the risk of adverse falls in these three situations of the domestic environment and frailty levels, as determined by the Edmonton Frail Scale, is not independent.

The Z + 4 test additionally confirms differences in percentages (inside the same answers) of those who answered that are aware of risk and those who are not, for those who are at least moderately frail.

To prove the hypothesis H2, we compared the percentages of those who recognize home fall risk because of non-adapted construction in different frailty categories according to the Edmonton Frail Scale. The following conclusions were determined:

The prevalence of perception of the risk of adverse falls and frailty levels as determined by the Edmonton Frail Scale is not independent in the cases of slippery floors (for subgroup C. only) and in the case of poorly installed or no fence at all (for subgroup C. only). It is also significant enough in the cases of movement in the domestic environment. In cases of inadequate, poor lighting, we found the contingency between perception of risk and frailty level quite significant for those already being hospitalized (*p*-value < 0.06).

In Table 6, there are given also results of the Z + 4 test of the difference in percentage of answers: p’(Yes)-q’(No) about perception of risk regarding the home construction and installations for moderate and severe frail older adults among the same answers “yes” or “no”. From there, we may conclude the following:

- In case of uneven, excessively low or high stairs, the percentage of those answering “yes” is significantly higher than those answering “no” only for subgroup C. at z-value = 1.30;
- In case of slippery floors, the percentage of those answering “yes” is significantly higher than those answering “no” only for subgroup C. at z-value = 3.054, but for subgroup H. only at z-value = 1.329;
- In case of unmarked edges, the percentage of those answering “yes” is significantly higher than those answering “no” only for subgroup H. at z-value = 1.545, but for subgroup C., it was not significant;
- In case of poorly installed or no fence at all, the percentage of those answering “yes” is significantly higher than those answering “no” only for subgroup H. at z-value = 5.823, but for subgroup C., it was not significant.

The results obtained strongly support the need for individualized adaptation of the living space (home) according to the level of frailty of the user. Smart technologies, ambient intelligence, ergonomic design, and barrier-free spaces should be a priority in creating a safe home environment, especially for older adults with moderate and severe frailty. The results provide a scientific basis for the development of a home risk assessment model based on an objective functional assessment of the user.

The dependence between the prevalence of perception of the risk of adverse falls in the outdoor environment between those who fell so hard that they required hospital treatment and those older adults who had never encountered such a problem related to falls is, in some situations, significant (H3). These conclusions were verified by analyzing the data presented in Table 7, Table 8, Table 9 and Table 10.

In the following situations of the outdoor environment, we found significant differences in the distribution of frailty regarding the prevalence of perception of the risk of adverse falls, at least in one subgroup:

- Going to a store, post office, bank, or medical facility;
- Using transport infrastructure;
- Insufficiently lit sidewalks and parks where walking;
- Walking on sidewalks and parks;
- Maintained and not slippery and uneven.

Therefore, we may conclude the following:

1. There is a high significance of dependency between frailty levels as determined by the Edmonton Frail Scale and the prevalence of perception of safety in the outdoor environment in general (going to a store, post office, bank, or medical facility) for the older adults who have been hospitalized—subgroup H. (*p*-value < 0.000002). The significance of this dependency in the subgroup C. is lower, *p*-value < 0.0007.
2. There is also significant dependency between frailty levels as determined by the Edmonton Frail Scale and the prevalence of perception of safety in the transport for the older adults who have not been hospitalized (subgroup C.) due to a prevalence of safety in transportation (*p*-value < 0.00001), but there is no significance of this dependency in the subgroup H. (*p*-value > 0.14).
3. Chi-square test of dependency shows that there is a certain dependency between the prevalence of perception of hazard and frailty in the subgroup C. (*p*-value < 0.0003), and in the subgroup H. (*p*-value < 0.0065) in case of insufficiently lit sidewalks and parks where they are walking.
4. Test of dependency shows that there is a certain dependency between the prevalence of perception of hazard and frailty in the subgroup C. (*p*-value < 0.05), and in the subgroup H. (*p*-value < 0.00001) when assessing how well-maintained are sidewalk is, how slippery or uneven it is.

The results, which we generalize to the entire population of the Zagreb region, do not include severely demented residents (less than 1% of the total population).

In further research, it would be advisable also to differentiate and compare the answers of patients enrolled in research during the acute phase and others (during rehabilitation or being discharged).

## 5. Limitations

Several limitations should be acknowledged when interpreting the findings.

1. The research design was cross-sectional. Longitudinal studies would be necessary to determine whether an increase in frailty precedes greater risk perception or whether heightened awareness contributes to behavioural changes that mitigate future falls.
2. The sample is geographically limited to the Zagreb region, which may constrain the generalizability of the results to international contexts.
3. Both the frailty level and the perception of risk were self-reported. Older adults might underestimate or overestimate their exposure to risks.
4. The environmental assessment relied on subjective perceptions rather than objective measurements. As a result, the study reflects perceived rather than actual environmental risk factors. Integrating objective spatial and infrastructural data would enhance the validity of future analyses.
5. Differences in health literacy, access to healthcare, and exposure to preventive programmes might influence the results.
6. In the future, we should explore in depth the qualitative dimension of lived experiences with environmental hazards.

Future research should build upon these findings by conducting longitudinal, multi-regional, and mixed-method investigations. In further research, it would be advisable also to differentiate and compare the answers of patients enrolled in research during the acute phase and others (during rehabilitation or being discharged).

## 6. Conclusions

The results of this study strongly confirm the existence of a complex and multidimensional relationship between the level of frailty and the perceived risks of falls in the daily life of older adults. Although more than half of the respondents from subgroup C. and quarte from subgroup H. do not show signs of frailty or show mild sensitivity, it is worrying that more than a quarter from C. and nearly half from H are on the spectrum of higher functional frailty (moderate and severe frail), which makes them susceptible to unwanted falls, bodily injury, loss of independence and possible institutionalization. It is in the group of the population 65+ that a higher frequency of those who reported obstacles was found in indoor spaces (kitchens, stairs, slippery surfaces like in bathrooms and elsewhere) and outdoors (pavements, access to public institutions, lighting), which indicates the need for proactive, targeted, and adapted interventions. Important statistical associations between frailty levels and risk perception confirm the importance of frailty assessment in planning measures to prevent unwanted falls. Older people not only face physical challenges, but a risky environment also has a negative impact on their independence, mental health, and social inclusion. The results of this research show the need for a holistic approach to preventing falls in older adults. It is crucial to introduce a mandatory home environment assessment as an integral part of geriatric care for older people who are at increased risk of adverse falls. We must especially focus on adapting the home and their living space outside the home for those older adults who have already experienced serious falls and been hospitalized, and others who are moderate or severely frail.

In addition, it is important to enable the adaptation of apartments and houses with subsidized programmes that include the installation of fences, anti-slip floors, and functionally adapted bathrooms. Improving community infrastructure, especially in rural areas, including flat and accessible sidewalks, ramps, adequate public lighting, and the removal of physical barriers, is an important factor in reducing the risk of unwanted falls.

In further research, we should study whether there are differences between urban and rural areas, continuing studies of authors from the list [35,36,37,38,39,40,41,42]. Also, we should consider how to educate the older adults, their caregivers, and family members to be able to recognize the risky situations. Encouraging safe behaviour is also crucial, because many of them are not aware of the risk of falls before experiencing them. Preventing the decline of the older population cannot only be the responsibility of the health system but also requires active and continuous cooperation with social activities in the design and implementation of local measures and policies. Based on the results of this study, it is recommended to develop a national strategy for the prevention of unwanted falls of older adults, which will consider the specific needs of people with different degrees of frailty, as well as the actual infrastructural conditions in which these people live. In this way, such approaches not only improve the quality of life of the older cohorts but also contribute to the sustainability of the health system, reduce the cost of treating injuries, and strengthen the social inclusion of society. There is a clear contingency between the degree of frailty and the perception of the risk of adverse home and community falls; therefore, identifying and removing infrastructure barriers, adapting space, and educating users are key elements of a comprehensive strategy to prevent unwanted falls of older adults. Investing in infrastructure and public policies that recognize the needs of older citizens can have long-term positive effects on population health and the sustainability of health systems; therefore, the results of this research can serve as a basis for the development of targeted public health interventions and strategies.

## Figures and Tables

**Table 1:** Distribution of frailty in samples by the Edmonton Frail Scale.

		N (Community) C.	N (Hospital) H.
RESULT	No frailty	77	34
	Sensitive	36	35
	Mild frailty	31	40
	Moderate frailty	36	39
	Severe frailty	20	52
	Sum	200	200

**Table 2:** Distribution of respondents by frailty according to whether they belong to the urban or rural population.

		No Frailty		Sensitive		Mild Frailty		Moderate Frailty		Severe Frailty		
		N	%	N	%	N	%	N	%	N	%	Sum
Do you live in	city	98	46.88	32	15.31	40	19.13	25	11.96	14	6.69	209
	countryside	84	44.21	39	20.52	35	18.42	20	10.52	12	6.31	190
	Sum	182	45.61	71	17.79	75	18.79	45	11.27	26	6.51	399

Source: Own research 2024, 2025.

**Table 3:** Distribution of persons being hospitalized (H.) and those from the Zagreb region who had never been hospitalized because of a fall (C.), regarding recognition of home fall hazards by different frailty categories according to the Edmonton Frail Scale.

		Not Frail		Sensitive		Mild Frail		Moderate Frail		Severe Frail		
		N	%	N	%	N	%	N	%	N	%	Sum
Recognition of a fall hazard that is present in their home: entering or leaving their home	C. Yes	11	14.3%	7	19.4%	8	25.8%	7	19.4%	6	30.0%	39
	C. No	66	85.7%	29	80.6%	23	74.2%	29	80.6%	14	70.0%	161
	C. Sum	77		36		31		36		20		200
					hospitalized							
	H. Yes	7	20.6%	8	22.9%	13	32.5%	12	30.8%	17	32.7%	57
	H. No	27	79.4%	27	77.1%	27	67.5%	27	69.2%	35	67.3%	143
	H. Sum	34		35		40		39		52		200
Recognition of a fall hazard that is present in their home: moving around their home	C. Yes	16	20.8%	5	13.9%	8	25.8%	4	11.1%	9	45.0%	42
	C. No	61	79.2%	31	86.1%	23	74.2%	32	88.9%	11	55.0%	158
	C. Sum	77		36		31		36		20		200
					hospitalized							
	H. Yes	7	20.6%	8	22.9%	16	40.0%	13	33.3%	23	44.2%	67
	H. No	27	79.4%	27	77.1%	24	60.0%	26	66.7%	29	55.8%	133
	H. Sum	34		35		40		39		52		200
Recognition of a fall hazard that is present in their home: climbing up and down stairs	C. Yes	36	46.8%	22	61.1%	18	58.1%	28	77.8%	13	65.0%	117
	C. No	41	53.2%	14	38.9%	13	41.9%	8	22.2%	7	35.0%	83
	C. Sum	77		36		31		36		20		200
					hospitalized							
	H. Yes	20	58.8%	20	57.1%	19	47.5%	23	59.0%	35	67.3%	117
	H. No	14	41.2%	15	42.9%	21	52.5%	16	41.0%	17	32.7%	83
	H. Sum	34		35		40		39		52		200
Recognition of a fall hazard that is present in their home: use of the kitchen	C. Yes	5	6.5%	5	13.9%	3	9.7%	9	25.0%	6	30.0%	28
	C. No	72	93.5%	31	86.1%	28	90.3%	27	75.0%	14	70.0%	172
	C. Sum	77		36		31		36		20		200
					Hospitalized							
	H. Yes	1	2.9%	4	11.4%	3	7.5%	4	10.3%	7	13.5%	19
	H. No	33	97.1%	31	88.6%	37	92.5%	35	89.7%	45	86.5%	181
	H. Sum	34		35		40		39		52		200

Source: Own research 2024, 2025.

**Table 4:** Dependence between the perception of risk of falling at home and various frailty categories according to the results of the Chi-square and Z + 4 tests for differences in percentages for moderate and severe frail persons.

		Chi-Square Test	Z + 4 Test of Differences in Percentages Between YES and NO for Moderate and Severe Frailty		
Exposure to Risk	Groups	*p*-Value	p’(Yes)-p’(No)	z-Value	*p*-Value
Entering or leaving the home difference between YES and NO	From community C.	>0.4	0.0715	0.88	>0.1
Entering or leaving the home difference between YES and NO	From hospital H.	>0.6	0.0740	0.97	>0.1
Entering or leaving the home difference between C. and H.	Yes, between C. and H.	>0.2	(0.1670) *	1.71	<0.05
Moving around their home difference between YES and NO	From community C.	<0.04	0.0432	0.55	>0.1
Moving around their home difference between YES and NO	From hospital H.	<0.1	0.1214	1.66	<0.05
Moving around their home difference between C. and H.	Yes, between C. and H.	>0.1	(0.0966) *	1.18	>0.1
Climbing up and down stairs, the differences between YES and NO	From community C.	<0.04	0.1647	2.71	<0.05
Climbing up and down stairs, the differences between YES and NO	From hospital H.	>0.4	0.0958	1.37	<0.1
Climbing up and down stairs, differences between C. and H.	Yes, between C. and H.	<0.05	(0.0470) *	0.68	>0.1
Using their kitchen, the differences between YES and NO	From community C.	<0.02	0.2919	3.06	<0.05
Using their kitchen, the differences between YES and NO	From hospital H.	>0.5	0.1288	1.15	>0.1
Using their kitchen, differences between C. and H.	Yes, between C. and H	>0.5	(−0.0910) *	−0.93	>0.1

C.: from community; no experience of hospitalization; H.: experienced a fall and was hospitalized. * Differences between C. (Yes) and H. (Yes) distributions. Source: Own research 2024–2025.

**Table 5:** Comparison of the percentage of those who recognize home fall risk because of non-adapted construction in different frailty categories according to the Edmonton Frail Scale.

Question: Which of the Listed Fall Hazards Is Present in Your Home		Not Frail		Sensitive		Mild Frailty		Moderate Frailty		Severe Frailty		
		N	%	N	%	N	%	N	%	N	%	N
uneven, excessively low, or high stairs	C. Yes	22	28.6	13	36.1	15	48.4	16	44.4	9	45.0	75
	C. NO	55	71.4	23	63.9	16	51.6	20	55.6	11	55.0	125
	C. Sum	77		36		31		36		20		200
uneven, excessively low, or high stairs	H. Yes	18	52.9	19	54.3	16	40.0	19	48.7	24	46.2	96
	H. NO	16	47.1	16	45.7	24	60.0	20	51.3	28	53.8	104
	H. Sum	34		35		40		39		52		200
slippery floors	C. Yes	20	26.0	15	41.7	8	25.8	17	47.2	13	65.0	73
	C. NO	57	74.0	21	58.3	23	74.2	19	52.8	7	35.0	127
	C. Sum	77		36		31		36		20		200
slippery floors	H. Yes	13	38.2	16	45.7	23	57.5	22	56.4	30	57.7	104
	H. NO	21	61.8	19	54.3	17	42.5	17	43.6	22	42.3	96
	H. Sum	34		35		40		39		52		200
unmarked edges	C. Yes	11	14.3	5	13.9	6	19.4	7	19.4	4	20.0	33
	C. NO	66	85.7	31	86.1	25	80.6	29	80.6	16	80.0	167
	C. Sum	77		36		31		36		20		200
unmarked edges	H. Yes	6	17.6	6.0	17.1	8.0	20.0	11.0	28.2	14.0	26.9	45
	H. NO	28	82.4	29	82.9	32	80.0	28	71.8	38	73.1	155
	H. Sum	34		35		40		39		52		200
poorly installed or no fence at all	C. Yes	17	22.1	8	22.2	4	12.9	8	22.2	6	17	43
	C. NO	60	77.9	28	77.8	27	87.1	28	77.8	14	60	157
	C. Sum	77		36		31		36		20	77	200
poorly installed or no fence at all	H. Yes	3	8.8	6.0	17.1	7.0	17.5	15	38.5	30	3	61
	H. NO	31	91.2	29	82.9	33	82.5	24	61.5	22	31	139
	H. Sum	34		35		40		39		52	34	200
inadequate or poor lighting	C. Yes	9	11.7	7	19.4	5	16.1	6	16.7	3	15.0	30
	C. NO	68	88.3	29	80.6	26	83.9	30	83.3	17	85.0	170
	C. Sum	77		36		31		36		20		200
inadequate or poor lighting	H. Yes	2	5.9	8	22.9	11	27.5	6.0	15.4	16	30.8	43
	H. NO	32	94.1	27	77.1	29	72.5	33	84.6	36	69.2	157
	H. Sum	34		35		40		39		52		200

Source: Own research 2024.

**Table 6:** Results of the Chi-square and Z + 4 test in the analysis of the contingency between the perception of the risk of falling in the home and different degrees of frailty categories according to the Edmonton Frail Scale.

Which of the Fall Hazards Is Present in Your Home:	CHISQ.TEST	Z + 4 Test of Difference (Yes-No) for Moderate and Severe Frailty			
	*p*-Value	p′ and q′ (4 + 5)	p′-q′	SE	Z
Uneven, excessively low, or high stairs, C.		0.338	0.086	0.066	1.301
	>0.2	0.252			
Uneven, excessively low, or high stairs, H.		0.449	−0.013	0.069	−0.191
	>0.7	0.462			
Slippery floors, C.		0.413	0.204	0.067	3.054
	<0.006	0.209			
Slippery floors, H.		0.500	0.092	0.069	1.329
	>0.3	0.408			
Unmarked edges (especially door threshold), C.		0.343	0.071	0.086	0.820
	>0.9	0.272			
unmarked edges (especially door threshold), H.		0.553	0.126	0.082	1.545
	>0.6	0.427			
Poorly installed or no fence at all, C.		0.333	0.063	0.078	0.808
	>0.6	0.270			
Poorly installed or no fence at all.		0.730	0.397	0.068	5.823
	<0.001	0.333			
Inadequate or poor lighting, C.		0.313	0.033	0.088	0.382
	>0.8	0.279			
Inadequate or poor lighting, H.		0.511	0.071	0.084	0.849
	<0.055	0.440			

Source: Own research 2024, 2025.

**Table 7:** Comparison of the presence of barriers in the outdoor environment (going to shops, post office, bank, health facilities) according to the different categories of frailty when using the Edmonton Frail Scale.

		Not Frailty		Sensitive		Mild Frailty		Moderate		Severe Frailty		Sum
		N	%	N	%	N	%	N	%	N	%	N
Are there any obstacles when you go to a store, post office, bank, or medical facility?	C. Yes	13	17.0	12	33	13	0.4	18	0.5	8	0.4	64
	C. NO	64	0.8	24	0.7	18	0.6	18	0.5	12	0.6	136
	C. Sum	77		36		31		36		20		200
	Hospitalized											
	H. Yes	11	0.3	8	0.2	16	0.4	28	0.7	28	0.5	91
	H. NO	23	0.7	27	0.8	24	0.6	11	0.3	24	0.5	109
	H. Sum	34		35		40		39		52		200

**Table 8:** Prevalence of the opinion in the Zagreb region that the transportation infrastructure is adequate for the older adults and people with disabilities and does not pose a risk of falling.

		Not Frailty		Sensitive		Mild Frailty		Moderate		Severe Frailty		Sum
		N	%	N	%	N	%	N	%	N	%	N
The transport infrastructure in their town is suitable and does not pose a risk of falling.	C. Yes	41	53.2	20	55.6	15	48.4	12	33.3	5	25.0	93
	C. NO	36	46.8	16	44.4	16	51.6	24	66.7	15	75.0	107
	C. Sum	77		36		31		36		20		200
	Hospitalized											
	H. Yes	15	44.1	18	51.4	17	42.5	13	33.3	18	34.6	81
	H. NO	19	55.9	17	48.6	23	57.5	26	66.7	34	65.4	119
	H. Sum	34		35		40		39		52		200

**Table 9:** Comparison of perceptions of safety when the streets and parks they walk through are sufficiently lit.

		Not Frail	Sensitive	Middle Frail	Moderate	Severe	Total
Question		N1	N2	N3	N4	N5	N
The streets and parks they walk through are sufficiently lit.	C. Yes	56	24	19	17	10	126
	C. NO	21	12	12	19	10	74
	C. Sum	77	36	31	36	20	200
	H. Yes	21	24	22	23	24	114
	H. NO	13	11	18	16	28	86
	H. Sum	34	35	40	39	52	200

**Table 10:** Comparison of perceptions of safety regarding regularly maintained and not slippery and uneven sidewalks they move.

		Not Frail	Sensitive	Middle Frail	Moderate	Severe	Total
The Claim		N1	N2	N3	N4	N5	N
The sidewalk they move on is regularly maintained and not slippery or uneven.	C. Yes	45	11	11	16	9	92
	C. NO	32	25	20	20	11	108
	C. Sum	77	36	31	36	20	200
	H. Yes	18	23	16	18	16	91
	H. NO	16	12	24	21	36	109
	H. Sum	34	35	40	39	52	200

Source: Own research 2024, 2025.

## Data Availability

Data will be provided on request by the authors. This research is part of ongoing research project. Data will be published at the end of the project.
